# High pretreatment systemic immune-inflammation index values are associated with diminished short-term success after temporomandibular joint arthrocentesis procedure

**DOI:** 10.1186/s12903-021-01899-0

**Published:** 2021-10-15

**Authors:** Efsun Somay, Busra Yilmaz

**Affiliations:** 1 Department of Oral and Maxillofacial Surgery, Faculty of Dentistry, Baskent University, Ankara, Turkey; 2 Department of Dentomaxillofacial Radiology, Faculty of Dentistry, Baskent University, Ankara, Turkey

**Keywords:** Temporomandibular joint arthrocentesis, Systemic immune-inflammation index, Temporomandibular joint disorder, Biomarker, Success

## Abstract

**Background:**

The systemic immune-inflammation index (SII) has been demonstrated to be a valid biomarker of a patient's immunological and inflammatory state, with the ability to accurately predict outcomes in a variety of disease conditions. In the absence of comparable studies, we intended to examine the relevance of pretreatment SII in predicting the success rates of temporomandibular joint arthrocentesis (TMJA) at 1-week, 1-month, and 6-month periods, defined as maximum mouth opening (MMO) > 35 mm and VAS ≤ 3.

**Methods:**

A sum of 136 patients with disc displacement without reduction (DDwo-red) who underwent TMJA was included. For each patient, pre-TMJA SII was calculated as; SII = Platelets × neutrophils/lymphocytes. Additionally, baseline MMO and VAS measurements were recorded for each patient. The success criteria of TMJA included MMO > 35 mm and VAS ≤ 3. The optimal pre-TMJA SII cutoff that predicts TMJA success was determined using receiver operating characteristic (ROC) curve analysis. The primary endpoint was the link between the pre-treatment SII and TMJA success (simultaneous achievement of MMO > 35 mm and VAS ≤ 3).

**Results:**

The median pre-TMJA jaw locking duration, maximum mouth opening (MMO), and visual analog score (VAS) were 7 days, 24 mm, and 8, respectively. The overall TMJA success rates were determined as 80.1%, 91.9%, and 69.1% at 1-week, 1-month, and 6-months, respectively. The results of ROC curve analysis exhibited the optimal SII cutoff at 526 (AUC: 67.4%; sensitivity: 66.7%; specificity: 64.2%) that grouped the patients into two subgroups: Group 1: SII ≤ 526 (N = 81) and SII > 526 (N = 55), respectively. Spearman correlation analysis revealed a strong inverse relationship between the pretreatment SII values and the success of TMJA 1-week (r_s_: − 0.83; *P* = 0.008) and 1-month, (r_s_: − 0.89; *P* = 0.03). Comparative analyses displayed that TMJA success rates at 1-week (87.7% vs. 69.1%; *P* = 0.008) and 1-month (96.2% vs. 80%; *P* = 0.03) were significantly higher in the SII ≤ 526 than SII > 526 group, respectively, while the 6-month results favored the SII ≤ 526 group with a trend approaching significance (*P* = 0.084).

**Conclusion:**

The current study's findings suggested the SII as a unique independent prognostic biomarker that accurately predicts treatment outcomes for up to 6 months.

*Trial registration* The results of this research were retrospectively registered.

## Background

Temporomandibular joint disorders (TMD) are a diverse group of diseases affecting the temporomandibular joint (TMJ), masticatory muscles, and related structures with an overall prevalence rate of 5% to 12% in the general population [1, 2]. Severe pre-auricular and masticatory area pain, joint sounds, and diminished mouth opening capacity embodies the most frequent TMD-related presenting symptoms [3]. The most certain intraarticular cause of TMD is disc displacement with (DDw-red) or without reduction (DDwo-red), which can cause severe joint degeneration and a sharp decline in quality-of-life (QoL) measures related to persistent chronic pain, impaired eating functions, limitation of normal jaw activities, difficulty in falling asleep, and resulting fatigue in DDwo-red patients [2, 4]. Blanco et al. concluded in the largest study ever conducted, with 1220 individuals, that TMD can induce significant pain sensations that impair QoL measurements. Furthermore, Bitiniene et al. showed in a comprehensive systematic review encompassing 12 research published between 2006 and 2016 that psychological and physical problems caused by TMD resulted in diminished patient QoL records [5, 6].

TMJ arthrocentesis (TMJA), with its 70% to 90% long-term success rate, is a relatively easy to perform and highly feasible minimally invasive procedure that efficiently reduces the complaints of DDwo-red patients who are resistant to conservative treatments (self-care measures, occlusal splint, and physical therapy) and drugs [7, 8]. However, literature related to the short-term success of TMJA is scant [9]. The reported prognostic factors for the TMJA procedure usually refer to the patient’s age, disease duration, pain severity, maximum mouth opening (MMO) capacity, and the presence of degenerative changes in the magnetic resonance imaging (MRI) scans. But sadly, emphasizing the compelling need for the identification of novel reliable prognosticators, accessible research results are often conflicting [10].

Another implied cause of TMD is local and chronic systemic inflammation, which manifests with the secretion of various biomarkers in the TMJ synovium, such as cytokines and growth factors [11]. Therefore, TMJA procedure is commonly used to defeat this inflammatory load in the joint cavity, while this procedure additionally promotes disc repair and repositioning by removing the fibrous tissues in DDwo-red patients [7, 8]. In this sense, it is of interest to identify TMD-specific inflammatory cytokines and develop specific treatments for this particular region [10]. For this purpose, Somay and Araz and Kaneyama et al. investigated the influence of pro-inflammatory cytokines and their values in synovial fluid on the success of TMJA, and declared that some inflammation and inflammatory mediators were extremely high in the synovial fluid, which meaningfully impacted the TMJA success rates [12, 13].

SII (Systemic Immune-Inflammation Index), a unique measure of the platelet, neutrophil, and lymphocyte counts, is a biomarker that mirrors the harmony between the patient’s inflammatory and immune status, irrespective of the underlying cause [14]. SII has recently been reported to be effective in predicting the prognosis of many diseases, including osteoporosis, osteoporotic bone fractures, psoriatic arthritis, and Bell's palsy [15–17]. In a clinical study of 23 patients, Somay and Araz investigated the impact of the synovial fluid sIL-1RII, sTNF-αRI and sIL-6R concentrations on the success rates of TMJA procedure [12]. The authors reported that the higher sIL-6R concentrations were associated with significantly reduced TMJA success rates.

Nevertheless, to the best of our knowledge, SII has never been examined for its actual impact on the success rates of the TMJA procedure, despite the accessibility of clinical evidence indicating a considerable detrimental influence of inflammation on the success of the TMJA. Consequently, given that the primary goal of the TMJA procedure is to remove the inflammatory debris together with the presence of the above-mentioned positive clinical data [12], we sought to investigate the prognostic significance of the pre-treatment SII on the short-term success of the TMJA procedure.

## Materials and methods

### Ethical approval

The present retrospective study was designed, authorized and approved by the institutional review board of Baskent University Medical Faculty (Project no: D-KA19/19) before the compilation of any persistent data, according to the Declaration of Helsinki. The eligible patients provided signed informed consent before the initiation of all dental and medical procedures either themselves or legitimately charged caregivers for acquisition and analysis of the patients’ sociodemographic, dental, and medical records; blood samples, MRI scans, and publication of the outcomes. The results of this research were retrospectively registered and evaluated.

### Study population

We conducted a retrospective search of clinical, medical, and radiological records (MRI, Magnetron “Harmony” Siemens, Erlangen, Germany) maintained by the Baskent University Adana Research and Practice Center Dentistry Clinic to identify ≥ 18 years old patients who underwent TMJA for TMD between September 2018 and July 2020. The Diagnostic Criteria for Temporomandibular Joint Disorders (DC/TMD) were used to determine the absence/presence of TMD [2, 18]. To be eligible, all patients had to have a diagnosis of DDwo-red according to these criteria, the Visual Analog Scale (VAS) measured TMJ pain value of ≥ 4, MMO < 35 mm, and available pre-TMJA complete blood count tests. Patients presenting with TMJ pain who had failed two months of conservative treatment (self-care preventions, occlusal splints, physical therapy, and medications) were included. In order to prevent the unintentional biasing effect of baseline immune and inflammatory conditions and drug usage patients who presented with either of the muscle-related pains, systemic inflammatory conditions such as rheumatologic diseases, nephritic disorders, respiratory diseases, viral hepatitis, TMJ ankylosis, proven immune suppressive disorders, chronic inflammatory conditions such as pancreatitis, previous TMJ surgery, history of trauma, and missing natural upper and/or lower central incisors were excluded from the present analysis. Patient complaints, like the presence of pain, restricted mouth opening, masticatory muscle tenderness, jaw deviation during mouth opening, bruxism, were all noted, and also the types of diagnosis indicated by MRI examination were grouped. The jaw locking duration was measured for each patient with limited mouth opening was also recorded, which was defined as the interim delaying the mouth opening and prohibiting eating functions according to the Mandibular Function Impairment Questionnaire [19]. All patients received conservative medications and physical therapy as a norm before the TMJA procedure, with TMJA being offered only for resistant cases.

### Preoperative assessments

For each eligible patient, the age, gender, presence of bruxism, jaw deviation during mouth opening, and muscle tenderness were noted before the TMJA procedure. Bruxism was diagnosed when the patient had a history of tooth clenching and grinding occurring ≥ 3 nights per week at the past 6-months [20], the experience of morning stiffness, presence of tooth wear [21], and also the appearance of linea alba in the cheek [22].

The preoperative clinical evaluations incorporated the pain assessments with the VAS [23] and MMO measurements. We used the original version of the VAS assessment tool, which typically consists of a 10-cm line describing the maximum and minimum measurement size values, where the two extremes 0 and 10 represent no pain and unbearable pain, individually. The same competent dentomaxillofacial radiologist (BY) completed the VAS assessments for each patient [24]. Likewise, the MMO was assessed preoperatively and at 1-week, 1-month, and 6-month intervals following the TMJA procedure by the same dentomaxillofacial surgeon (ES). The VAS test was carried out at the same time intervals as the MMO measurements. The 6-month results were specifically obtained to examine the TMJA procedure's short-term efficacy. The treatment was considered successful if the MMO was > 35 mm and the VAS was less ≤ 3 simultaneously [7].

### Measurement of maximum mouth opening (MMO)

We have utilized the range of motion scale Therabite® (Atos Medical AB, Hörby, Sweden) to measure MMOs since it enables easy and direct measurement and decreases the risk of procedure-related infection due to its disposable characteristic [25]. Each patient was instructed to open her/his mouth as widely as practically feasible to measure the distance between the top borders of one of the lower central incisors and the lower edge of one of the upper central incisors, with the Therabite® range of motion scale being inserted in the mouth. As a standard, MMO measurements were performed three times per session, with the mean MMO being computed as the average of the three successive measurements.

### Arthrocentesis procedure

All patients underwent a single-session TMJA procedure performed by the same oral and maxillofacial surgeon (ES). After measuring the patient's MMO, the preauricular skin and ear regions were prepared by cleaning with a topical antiseptic solution (Povidone-Iodine 10% w/v), and the surgical area was confined with a sterile cover. To block the auriculotemporal nerve, we applied 1–2 ml of a local anesthetic solution [Ultracain® DS Forte 40 mg/ml articaine HCL, 0.0012 mg/ml epinephrine (Sanofi-Aventis, Frankfurt, Germany)] over the preauricular region and into the superior joint space (SJS). As previously described by Nitzan et al., a line was drawn from the most posterior and central point on the tragus to the lateral canthus of the eye by a sterile skin pen [7]. The first entry point was marked 10-mm anterior of the tragus and 2-mm below this line, while the second entry point was 20-mm anterior and 8-mm below this line. Then, a 21‐gauge needle was inserted into the SJS at the glenoid fossa through the first point, and approximately 2–3 mL of Ringer's solution was pumped ten times to expand the SJS. A second 20‐gauge needle was then inserted from the second entry point to enable the free flow of the irrigation solution in the SJS. Lavaging with free outflow under high pressure and with a minimum of 400 ml Ringer's solution was considered regarded as effective irrigation [7, 26]. All patients were advised for a soft diet, hot pad application, and passive stretching exercises for a week following surgery. Analgesics and myorelaxants were prescribed as needed. We standardly prescribed an occlusal splint for at least one month following surgery to prevent bruxism and associated problems.

### Systemic immune-inflammation index (SII) assessment

We ran Hu’s original equation to calculate the pre-TMJA SII values for each patient: SII = [Platelets × (Neutrophils/Lymphocytes)], by using the routine complete blood count tests performed on the TMJA day [27].

### Statistical analysis

The primary endpoint was the connection between the pretreatment SII values and the TMJA success. Medians and ranges were calculated to describe continuous variables, while categorical variables were expressed with percentage frequency distributions. Chi-square test, Student's t-test, or Spearman correlation analyses and related r_s_ values were employed to compare the patient groups, as indicated. Receiver operating characteristic (ROC) curve analysis was employed to define ideal cutoff(s) that may group the whole study into two distinctive outcome groups, such as the pre-TMJA SII measures. All comparisons were two-tailed, and any *P* value < 0.05 was considered significant.

## Results

Our retrospective database search yielded a sum of 136 patients who had underwent TMJA procedure. As summarized in Table 1, the median age was 34 years (range: 18–59), with a female predominance (77.9%). Pain (47.8%) and difficulty in mouth opening (33.1%) accounted for the majority (80.9%) of the presenting complaints. The median jaw locking duration, pre-TMJA MMO, pre-TMJA VAS were 7 days [95% confidence interval (CI): 2.8–14 days], 24 mm (95% CI: 17.8–28.4 mm), and 8 (95% CI: 6.7–8.7), respectively. Presenting patient and TMD characteristics including the muscle tenderness, deviation during mouth opening, and bruxism were as shown in Table 1. Of note, 50.7% of patients had bilateral DDwo-red (Table 1).

**Table 1:** Baseline patient and temporomandibular disorder characteristics for all patients group and per systemic immune-inflammation index groups

Characteristics	All patients (N = 136)	SII ≤ 526 (N = 81)	SII > 526 (N = 55)	*P* value
Median age, years (range)	34 (18–59)	35 (20–59)	33 (19–58)	0.81
Gender, N (%)				
Female	106 (77.9)	64 (79.0)	42 (76.4)	0.86
Male	30 (22.1)	17 (21.0)	13 (23.6)	
Presenting complaints, N (%)				
Pain	65 (47.8)	39 (48.1)	26 (47.3)	0.38
Difficulty in mouth opening	45 (33.1)	29 (35.8)	16 (29.1)	
Both	26 (19.1)	13 (16.1)	13 (23.6)	
Median jaw locking duration, day (95% CI)	7 (2.8–14)	8 (4–13)	6 (3–14)	0.22
Median pre-TMJA MMO, mm (95% CI)	24 (17.8–28.4)	22.6 (18.2–28.1)	24.7 (19.1–28.4)	0.34
Median pre-TMJA VAS (95% CI)	8 (6–9)	8 (7–9)	8 (6–9)	0.94
Muscle tenderness, N (%)				
Present	27 (19.9)	16 (19.8)	11 (20.0)	0.91
Absent	109 (80.1)	65 (80.2)	44 (80.0)	
Deviation during mouth opening, N (%)				
Present	44 (32.4)	28 (34.6)	16 (29.1)	0.52
Absent	92 (67.6)	53 (65.4)	39 (70.9)	
MRI findings, N (%)				
Unilateral DDwo-red	67 (49.3)	37 (45.7)	30 (54.5)	0.41
Bilateral DDwo-red	69 (50.7)	44 (54.3)	25 (45.5)	
Bruxism, N (%)				
Present	62 (45.6)	40 (50.6)	21 (38.2)	0.19
Absent	74 (54.4)	40 (49.4)	34 (61.8)	

*CI* confidence interval, *DDwo-red* disc displacement without reduction, *mm* millimeter, *MMO* maximum mouth opening, *MRI* magnetic resonance imaging, *SII* systemic immune-inflammation index, *TMD* temporomandibular joint disorder, *TMJA* temporomandibular joint arthrocentesis, *VAS* visual analog scale

**Fig. 1:**
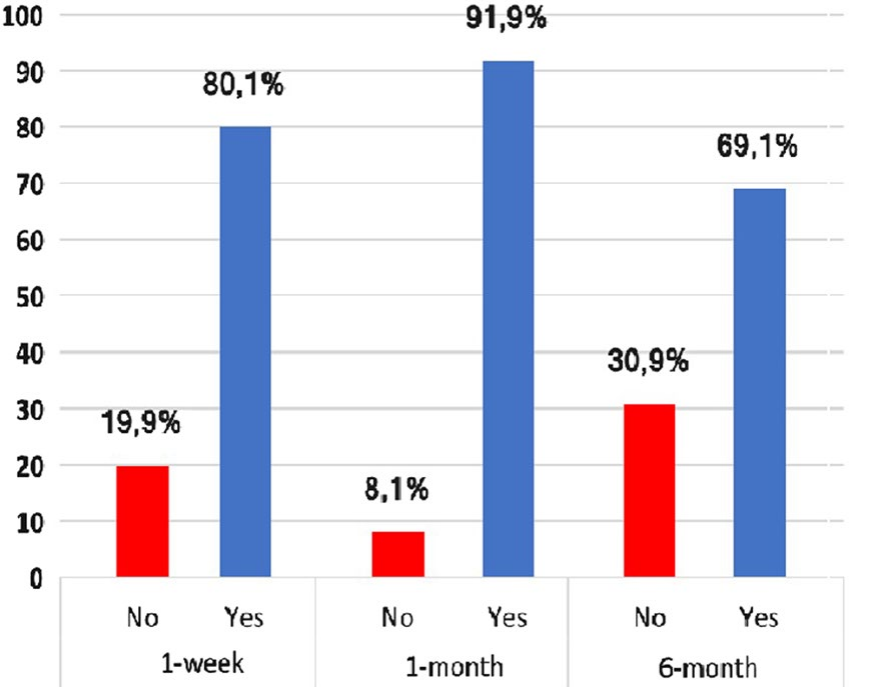
Overall success of TMJA at 1-week, 1-month, and 6-months evaluations

**Fig. 2:**
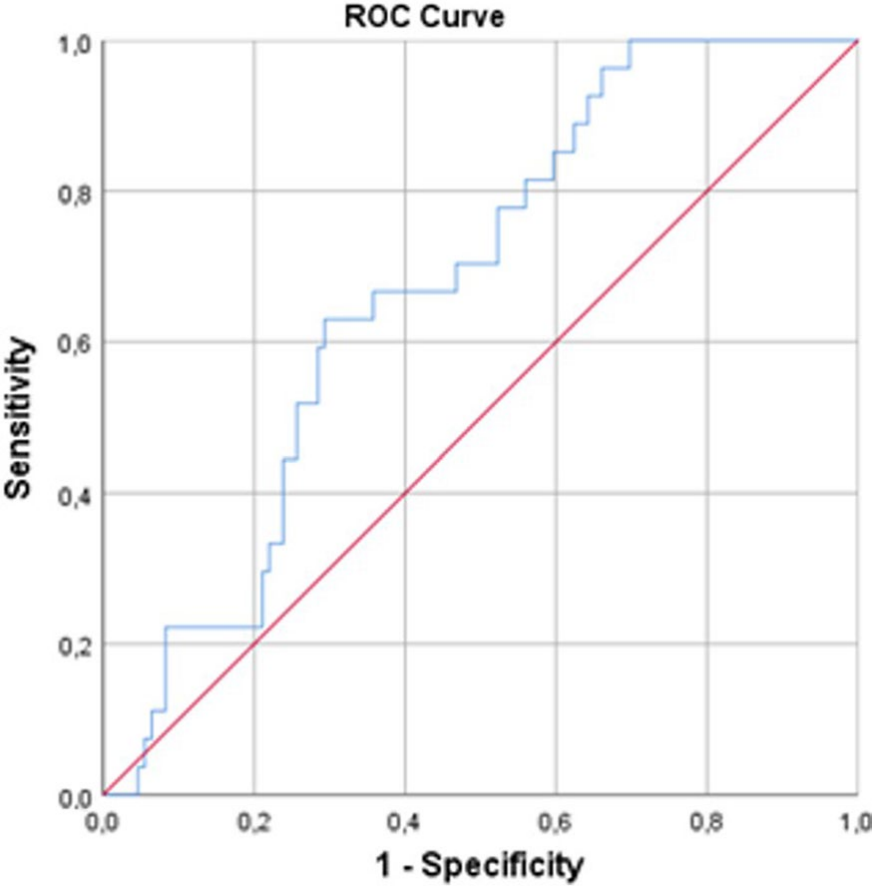
Results of ROC analysis evaluating the relationship between the pretreatment SII measures and TMJA success

Overall, we found that the 1-week, 1-month, and 6-month TMJA success rates were 80.1% at, 91.9% at 1-month, and 69.1%, respectively, per success criteria defined as MMO > 35 mm and VAS ≤ 3 (Fig. 1). We used ROC curve analysis to reveal the possible connections between the pre-treatment SII levels and TMJA success at 1-week, 1-month, and 6-month intervals. The ROC curve analysis results exhibited the optimal cutoff value at 526 (Area under the curve (AUC): 67.4%; sensitivity: 66.7%; and specificity: 64.2%) for 1-week, 527 (AUC: 66.2%; sensitivity: 65.8%; and specificity: 64.0%), and 524 (AUC: 65.1%; sensitivity: 64.3%; and specificity: 64.1%) for 6-months, respectively. Since the three cutoff values were so close, we utilized the 526 as the common cutoff to separate patients into two groups for all time-dependent TMJA success evaluations (Fig. 2): Group 1: SII ≤ 526 (N = 81) and SII > 526 (N = 55), respectively. Spearman correlation analysis revealed a strong and significant inverse relationship between the pre-treatment SII values and the TMJA success at 1-week and 1-month (r_s_: − 0.83; *P* = 0.008, r_s_: − 0.89; *P* = 0.03), with an additional trend favoring the SII ≤ 526 group for the TMJA success at 6-month (r_s_ = − 0.42; *P* = 0.086) evaluations (Table 2). Baseline patients and disease characteristics were almost evenly distributed between the two SII cohorts with no statistically significant differences between them (Table 1). As shown in Table 2, our comparative analyses displayed that the rates of TMJA success for 1-week (87.7% vs. 69.1%; *P* = 0.008) and 1-month (96.2% vs. 80%; *P* = 0.03) were significantly higher in the SII ≤ 526 group than its SII > 526 counterparts, respectively. Although the difference between the success rates of the two SII groups could not reach statistical significance at 6-months evaluations, we observed a strong trend approaching statistical significance favoring the SII ≤ 526 over the SII > 526 group (74.1% vs. 60%; *P* = 0.084).

**Table 2:** TMJA success for the per SII status

TMJA success	All patients (N = 136)	SII ≤ 526 (N = 81)	SII > 526 (N = 55)	*P* value
1-week, N (%)				
Yes	109 (80.1)	71 (87.7)	38 (69.1)	0.008
No	27 (19.8)	10 (12.3)	17 (30.9)	
1-month, N (%)				
Yes	122 (89.7)	78 (96.3)	44 (80.0)	0.03
No	14 (10.3)	3 (3.7)	11 (20.0)	
6-month, N (%)				
Yes	99 (72.8)	60 (74.1)	33 (60.0)	0.084
No	43 (27.2)	21 (25.9)	22 (40.0)	

*TMJA* temporomandibular joint arthrocentesis, *SII* systemic immune-inflammation index

**Fig. 3:**
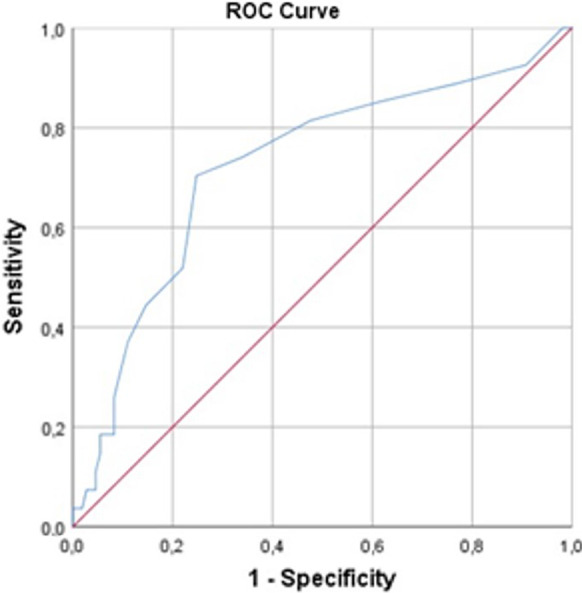
Results of ROC analysis evaluating the relationship between the pretreatment jaw locking duration and TMJA success

We further sought for the presence of additional relevant cutoff(s) for other covariates which may alter the TMJA success rates significantly in favor of one group (Table 3). Our search with ROC curve analysis revealed a significant cutoff uniquely for the jaw locking duration at a threshold of 7.5 days (AUC: 72.2% sensitivity: 74.1%; and specificity: 66.1%) (Fig. 3). Comparative analyses displayed that the rates of TMJA success were significantly higher in patients presenting with a jaw locking duration < 8 days at 1-week (66% vs. 34%; *P* < 0.001), 1-month (61.5% vs. 38.5%; *P* = 0.003), and 6-month (74.2% vs. 25.8%; *P* < 0.001) group than their ≥ 8 days counterparts.

**Table 3:** TMJA success per presenting patient and TMD characteristics

Characteristic	TMJA success 1-week		*P* value	TMJA success 1-month		*P* value	TMJA success 6-month		*P* value
	Yes	No		Yes	No		Yes	No	
Muscle tenderness, N (%)									
Yes	24 (22.0)	3 (11.1)	0.28	25 (20.5)	2 (14.3)	0.74	16 (17.2)	11 (25.6)	0.26
No	85 (78.0)	24 (88.9)		97 (79.5)	12 (85.7)		77 (82.8)	32 (74.4)	
Jaw deviation during mouth opening, N (%)									
Yes	34 (31.2)	10 (37.0)	0.65	39 (32.0)	5 (35.7)	0.77	26 (28.0)	18 (41.9)	0.12
No	75 (68.8)	17 (63.0)		83 (68.0)	9 (64.3)		67 (72.0)	25 (58.1)	
Types of MRI findings, N (%)									
Unilateral DDwo-red	51 (46.8)	16 (59.3)	0.27	60 (49.2)	7 (50.0)	0.66	45 (48.4)	22 (51.2)	0.86
Bilateral DDwo-red	58 (53.2)	11 (40.7)		62 (50.8)	7 (50.0)		48 (51.6)	21 (48.8)	
Bruxism, N (%)									
Yes	53 (48.6)	9 (33.3)	0.20	57 (46.7)	5 (35.7)	0.57	44 (47.3)	18 (41.9)	0.58
No	56 (51.4)	18 (66.7)		65 (53.3)	9 (64.3)		49 (52.7)	25 (51.1)	
Jaw locking duration, N (%)									
≥ 8	37 (34.0)	20 (74.1)	0.001	47 (38.5)	10 (71.4)	0.023	24 (25.8)	33 (76.7)	0.001
< 8	72 (66.0)	7 (25.9)		75 (61.5)	4 (28.6)		69 (74.2)	10 (23.3)	
Median Pre-TMJA MMO (mm), N (%)									
≤ 24	57 (52.2)	14 (51.9)	0.98	61 (50.0)	10 (71.4)	0.16	45 (48.4)	26 (60.5)	0.20
> 24	52 (47.8)	13 (48.1)		61 (50.0)	4 (28.6)		48 (51.6)	17 (39.5)	

*mm* millimeter, *MMO* maximum mouth opening, *MRI* magnetic resonance imaging, *TMD* temporomandibular joint disorder, *TMJA* temporomandibular joint arthrocentesis, *VAS* visual analog scale

## Discussion

The results of current retrospective research examining the impact of pre-TMJA values on the success rates of the TMJA procedure discovered that the pre-TMJA SII > 526 was independently associated with significantly lower 1-week (*P* = 0.008) and 1-month (*P* = 0.03) TMJA success rates, with an additional trend approaching significance at 6-months (*P* = 0.084) assessments. Furthermore, the longer presenting jaw locking duration (≥ 8 days; *P* = 0.001 for 1-week, *P* = 0.023 for 1-month, and *P* = 0.001 for 6-months) was found to have a detrimental influence on the short-term TMJA success rates.

The local inflammation of DD is caused by an inflammatory process on the TMJ joint surface and synovial fluid [28]. McCain discovered that synovial hypervascularity was more common and coexisted with exacerbated local inflammation in DDwo-red TMJ patients than in DDw-red TMJ patients [29]. Likewise, Nitzan et al. and Murakami et al. found that the impotence of the condyle to slide during regular mouth opening was linked to inflammatory alterations on the joint surface [7, 30]. Although the precise mechanisms attaching local and systemic inflammation in TMD have yet to be determined, TMD cartilage degeneration has been reported to be caused by expanded local inflammation in individuals with systemic inflammatory diseases such as osteoarthritis, as evidenced by the presence of elevated levels of pro-inflammatory and inflammatory mediators in the TMJ joint space [11]. Because circulating pro-inflammatory mediators such as TNF-α and IL-6 have been shown to influence TMJA success [11, 31, 32], it is prudent to assume that other systemic biomarkers, such as the SII, could also be relevant in predicting TMJA outcomes, given that local inflammation invokes systemic inflammation.

The primary discovery of this study was the demonstration of the SII as a novel indicator for TMJA success, in addition to its known prognostic efficacy in a large variety of diseases [33–35]. Despite the apparent lack of previous results to objectively compare these first outcomes, we can still propose some insightful hypotheses by considering the critical actions of local and systemic inflammation on the success of TMJA, which is exhibited by the immune and inflammatory cell components of the unique SII formula: platelets (PLTs), neutrophils, and lymphocytes. Increased peripheral blood PLT count is considered a powerful indicator of the state of the continuously rising systemic inflammatory response, which can promote occlusion of small blood vessels with subsequent bony ischemia in the jawbones [36]. Neutrophils are the most numerous immune cells in the oral cavity, which assume vital roles in local/systemic immunity and inflammation with their phagocytotic, and reactive oxygen species plus cytokine/chemokine manufacturing and secreting functions [37]. Unlike the inflammatory neutrophils, lymphocytes are immune cells that migrate to the injured region to fight against the causes of inflammation [38]. Hence, as a result of elevated neutrophil and PLT numbers and reduced lymphocyte counts, enhanced systemic inflammation results in a high SII score. Although investigations that directly address this issue are needed, the lower TMJA success rates at all time points in the high SII group may be related to persistent systemic inflammation in this particular patient gathering.

We uncovered an influential link between high pre-TMJA SII values and short-term success of TMJA procedure, especially at 1-week and 1-month, with an extra trend approaching significance at 6-months. This latter result, however, might be attributed to the limited sample size. Furthermore, because SII lost its predictive relevance at 6-months, we assume that the effect of pre-TMJA SII on the success of the procedure was reduced as a result of the time-dependent resolution of the local inflammation. Given these results it is possible that the TMJA treatment might have totally cleared, or at least significantly reduced, the local inflammation and its systemic extension in less than six months of the procedure.

Former research has proposed jaw locking duration as a notable predictor of TMJA success in DDwo-red patients [7, 38, 39]. We likewise distinguished the jaw locking duration as a significant indicator of TMJA success at 1-week, 1-month, and 6-month evaluations. In support, Kaneyama et al. proclaimed that the longer jaw locking durations were associated with the presence of severe synovial inflammation and reduced TMJA success rates [13]. Although the time cutoff of 1-month in Sembronio and colleagues’ study was remarkably longer than our 7-days, the authors reported that the longer jaw locking duration was linked to a significantly reduced TMJA success rate (87.5% vs. 68.0% for > 1-month; P) at 1-year [40]. Although the precise causes are unknown, it is reasonable to deduce that the inflamed synovium and associated local and/or systemic inflammatory mediators may have prolonged jaw locking durations, resulting in decreased TMJA success [32].

Depending on the post-procedural evaluation periods, the overall success rates of the TMJA treatment for TMDs range from 70 to 91.9% [7, 41, 42]. Although the patients were not scheduled on planned intermediate controls, representing the highest of any TMJA success rates ever recorded, Nitzan et al. noted respective success rates of 91% to 95% at 4 to 14 months of follow-up [7]. On the other hand, Murakami [41] and Hosaka [42] reported respective 70% and 79% success rates at 6-month assessments, where our corresponding 69.1% seems to be almost identical to Murakami's 70%. The observation that the 91.9% success rate after 1-month was decreased to 69.1% in this research and 70% in Murakami's study at 6-months of the TMJA suggested a time-dependent reduction in procedural success [41]. Nevertheless, confirming that the TMJA is a legitimate therapeutic strategy for refractory TMDs, our 69.1% success rate at 6-months is still superior to the 55.9% documented for conservative therapies in a previous meta-analysis [43].

The present research is handicapped with several shortcomings. First, because they apply only to single institutional retrospective research with a relatively small cohort size, the observed results should be regarded as just hypothesis-generating. Second, even though the SII was a dynamic systemic biomarker with significant time-dependent variations, our SII measurements were based on a single time-point estimation acquired immediately before the TMJA treatment. Third, we may have lost an opportunity to uncover the complicated processes behind the link between a higher SII value and lower TMJA success rates since we did not assess additional inflammatory markers such as TNF-α, IL-6, and many others. As a result, future studies focusing on these key concerns may give helpful information regarding the real influence of pre-treatment SII values on the TMJA results of TMD patients.

## Conclusion

The results of our current retrospective cohort analysis of 136 DDwo-red patients indicate that high measures of pre-TMJA SII are associated with diminished TMJA success rates at 1-week, 1-month, and 6-months time points. Hence, if additional research can approve these results, SII, a novel inflammatory and immune marker that is inexpensive, easy to implement, and calculate, can serve as a reliable predictor of TMJA success in TMD patients.

## Data Availability

Data cannot be shared publicly because the data is owned and saved by Baskent University Medical Faculty. Data are available from the Baskent University Institutional Data Access/Ethics Committee (contact via Baskent University Ethics Committee) for researchers who meet the criteria for access to confidential data: contact address, adanabaskent@baskent.edu.tr.

## Abbreviations

SII: Systemic Immune-Inflammation Index
TMD: Temporomandibular joint disorders
TMJ: Temporomandibular joint
TMJA: TMJ arthrocentesis
DDw-red: Disc displacement with
DDwo-red: Disc displacement without reduction
MMO: Maximum mouth opening
MRI: Magnetic resonance imaging
DC/TMD: The Diagnostic Criteria for Temporomandibular Joint Disorders
VAS: Visual Analog Scale
SJS: Superior joint space
ROC: Receiver operating characteristic
AUC: Area under the curve
CI: Confidence interval
PLTs: Platelets
